# Health-related quality of life in children and adolescents with primary hypertension: a hospital-based cross-sectional study

**DOI:** 10.3389/fpsyg.2026.1763465

**Published:** 2026-06-24

**Authors:** Aslı Berivan Topçak, Melek Hande Bulut Demir, Sevgin Taner, Merve Tosyalı, Ahmet Keskinoğlu, Caner Kabasakal, İpek Kaplan Bulut

**Affiliations:** 1 Department of General Pediatrics, Ege University Faculty of Medicine, Izmir, Türkiye; 2 Department of Child and Adolescent Psychiatry, Ministry of Health Dr. Behcet Uz Pediatric Diseases and Surgery Training and Research Hospital, Izmir, Türkiye; 3 Department of Pediatric Nephrology, Ege University Faculty of Medicine, Izmir, Türkiye

**Keywords:** adolescents, children, hypertension, PedsQL, quality of life

## Abstract

**Introduction:**

Primary hypertension in childhood is increasingly recognized as an important health issue, but its impact on health-related quality of life (HRQoL) has not been well studied.

**Methods:**

In this cross-sectional study conducted at the Pediatric Nephrology Department of Ege University Medical Center between June and December 2021, the Pediatric Quality of Life Inventory™ (PedsQL) 4.0 was administered face-to-face to 22 children and adolescents with primary hypertension and 31 healthy controls, along with their parents. Data were analyzed using IBM SPSS version 26.

**Results:**

A total of 53 children and their parents were included. Compared with healthy controls, the primary hypertension group demonstrated significantly lower parent-reported physical health total scores, parent total PedsQL scores, and child physical health total scores. No statistically significant differences were observed between the groups in several other PedsQL subdomains. No significant differences were found in HRQoL scores according to BMI groups or treatment types.

**Conclusion:**

Children and adolescents with primary hypertension demonstrated lower health-related quality of life scores, particularly in physical health-related domains, compared with healthy controls. These findings suggest that primary hypertension may be associated with impaired quality of life in pediatric patients. Larger, multicenter, longitudinal studies are needed to validate these results.

## 1 Introduction

Cardiovascular diseases (CVD) are the leading cause of death worldwide, and hypertension is the most important risk factor for CVD (Vaduganathan et al., 2022; Dagenais et al., 2020). The prevalence of hypertension in children has been reported to be between 3.5 and 5%, while elevated blood pressure has been reported in approximately 2.2 to 3.5% of children (Falkner and Daniels, 2004). In particular, the increasing prevalence of childhood hypertension has become a significant public health concern (Hardy and Urbina, 2021; Noubiap et al., 2017; Song et al., 2019).

In 2017, the American Academy of Pediatrics Clinical Practice Guidelines introduced updated pediatric blood pressure definitions according to age, height, and sex. Currently, blood pressure is classified as normal, elevated, stage 1 hypertension, or stage 2 hypertension (Flynn et al., 2017). Hypertension is classified etiologically into primary and secondary forms. Primary hypertension is associated with factors such as obesity, genetic predisposition, sedentary lifestyle, and unhealthy dietary habits, and it is more commonly observed in children over 6 years of age (McNiece et al., 2007). In contrast, in younger children, 75–85% of hypertension cases are attributed to secondary causes (Gomes et al., 2011; Arar et al., 1994; Flynn et al., 2012).

Health-related quality of life (HRQoL) is a general indicator of health and well-being that reflects an individual’s perception of their well-being and their ability to perform daily activities (Bowling, 1991). Previous studies have shown that chronic pediatric conditions may negatively affect multiple domains of health-related quality of life, including physical, emotional, social, and school functioning (Ingerski et al., 2010). However, data specifically addressing HRQoL in children with primary hypertension remain limited. Existing evidence suggests that hypertension may impair quality of life through short- and long-term complications, physical symptoms, lifestyle modifications, and psychological stress (Katsi et al., 2017; Sang et al., 2021). In recent years, measures of health-related quality of life (HRQoL) have been increasingly developed and have begun to be utilized to some extent in pediatric clinical practice (Varni et al., 2005). In this study, we decided to use the Turkish version of the Pediatric Quality of Life Inventory™ (PedsQL) 4.0, which is known to be an effective tool for assessing quality of life in children (Varni et al., 2001; Uneri et al., 2008; Çakin Memik et al., 2007).

Accordingly, this study aims to assess the quality of life of children with hypertension and their parents, and to determine the factors associated with it.

## 2 Materials and methods

### 2.1 Study design and setting

This study was conducted by the Pediatric Nephrology Department of Ege University Medical Center between June and December 2021. Using a cross-sectional design, face-to-face surveys were administered to children and adolescents with primary hypertension and their parents. The quality of life of the patients was compared to that of a healthy control group consisting of children who presented for routine evaluations at the General Pediatrics Clinic of the same hospital. Approval was obtained from the local, non-invasive Clinical Research Ethics Committee (date: January 21, 2021, document no: E-99166796-050.06.04-2439577) and written informed consent was acquired from the parent or legal guardian of each study participant.

### 2.2 Participants

The study included 22 children and adolescents aged 5–18 years who were being followed with a diagnosis of primary hypertension at the Pediatric Nephrology Outpatient Clinic of Ege University and whose parents provided informed consent. Primary hypertension was defined as systolic or diastolic blood pressure above the 95th percentile for age, sex, and height, confirmed by at least three separate measurements (Lurbe et al., 2009). Participants were required to be physically and cognitively capable of independently completing the age-appropriate questionnaires, based on their clinical status and the judgment of their parents.

The control group consisted of healthy children and adolescents aged 5–18 years who had no chronic or psychiatric diseases and who presented to the General Pediatrics Outpatient Clinic of Ege University for routine health evaluations, along with their parents.

Patients with major chronic disorders that could independently affect blood pressure regulation or health-related quality of life, such as neurogenic bladder, neurogenetic disorders, and hereditary diseases, were excluded from the study. However, relatively common accompanying conditions including obesity, metabolic syndrome, attention-deficit/hyperactivity disorder, and autism spectrum disorder were not considered exclusion criteria.

### 2.3 Measures

In this study, the Turkish translation of the PedsQL™ 4.0 Scale was used (Varni et al., 2001; Uneri et al., 2008; Çakin Memik et al., 2007). The PedsQL consists of a total of 23 items divided into four subscales: physical functioning (eight items), emotional functioning (five items), social functioning (five items), and school functioning (five items). The scale has four different forms tailored to specific age groups: 2–4, 5–7, 8–12, and 13–18 years. Children rated how often each item had been a problem for them during the past month using a five-point Likert scale (0 = never a problem, 1 = almost never a problem, 2 = sometimes a problem, 3 = often a problem, 4 = almost always a problem). Scores range from 0 to 100, with higher scores indicating better health related quality of life. The total PedsQL score was calculated by summing the scores of all items and dividing by the number of items answered. The internal consistency of the PedsQL scale (Cronbach’s alpha = 0.70–0.89) and its clinical reliability are high (Varni et al., 2001). Cakin Memik et al. reported the validity and reliability of the Turkish version of the PedsQL for adolescents aged 8–12 and 13–18 years (Çakin Memik et al., 2007). Üneri et al. also validated the scale for use in children between 2 and 7 years of age (Uneri et al., 2008).

In the present study, the age-appropriate self-report forms of the PedsQL 4.0 Generic Core Scales for children and adolescents aged 5–18 years were completed by the children and adolescents themselves. The parent-reported forms were completed separately by the parents based on their own perceptions of their children’s health-related quality of life.

Selected sociodemographic and family-related characteristics were recorded because of their potential influence on psychosocial well-being and health-related quality of life. All patients received routine lifestyle and nutritional recommendations as part of standard clinical management for pediatric hypertension. However, the term “diet therapy” in the present study referred specifically to patients who received structured dietary counseling and/or formal dietary follow-up. Information regarding dietary adherence was assessed based on self-report obtained from the children/adolescents and their parents during face-to-face interviews.

### 2.4 Statistical analysis

The statistical analyses were conducted using IBM® SPSS® 26 software (SPSS Inc., Chicago, IL, USA). The variables’ normality was assessed through analytical techniques, like the Kolmogorov Smirnov and Shapiro–Wilk tests. Descriptive analyses were given using mean ± standard deviation for normally distributed variables, median (median), and IQR for non-normally distributed variables. The descriptive statistics involved the provision of frequency and percentage values for categorical variables pertaining to sociodemographic and clinical data. The t-test was employed to compare independent groups in the context of continuous data, specifically PedsQL scores, when the data exhibited a normal distribution. Conversely, the Mann–Whitney U test was utilized to compare binary groups, such as patient-control and child-adolescent, when the data did not conform to a normal distribution. In the dependent groups, the PedsQL scores of the parents and children were compared with the t-test in the dependent groups with normal distribution and with the Wilcoxon Signed Ranks test in the absence of normal distribution. Pearson or Fisher exact chi-square tests were used to compare categorical variables. Kruskal-Wallis analysis was used to compare groups of three, such as drug therapy, and the Bonferroni test was used for *post hoc* comparisons and to identify significant groups. The relationship between PedsQL and its sub-dimensions and other variables (urea, uric acid, and creatinine) was evaluated with Spearman’s correlation analysis. ROC analysis was used to determine the cut-off values, sensitivity-specificity values, and predictive variables in PedsQL and sub-dimension scores. The cases where the *p*-value was below 0.05 statistically were considered significant.

## 3 Results

### 3.1 Demographic and clinical characteristics

A total of 53 children and their parents were included in the study. Of these, 22 (41.5%) were patients and 31 (58.5%) were healthy controls. The children consisted of 28 boys (52.8%) and 25 girls (47.2%). Twelve participants (22.6%) were in the 8–12 years age group, while 41 participants (77.4%) were in the 13–18 years age group. The demographic, family-related, and psychosocial characteristics of the study groups are presented in Table 1. Significant differences were observed between the groups in terms of parental substance use, presence of siblings, and psychotropic medication use. Parental use of both cigarettes and alcohol and having siblings were markedly higher in the primary hypertension group, whereas psychotropic drug use was significantly more common in the control group. No significant differences were detected between the groups regarding age distribution, sex, or parental employment status (Table 1).

**Table 1:** Comparison of demographic, family, and psychosocial characteristics between the primary hypertension and healthy control groups.

Variables	Subgroups	Primary hypertension group n (%)	Healthy control group n (%)	*p*-value
Age group	8–12 years (child)	5 (22.7)	7 (22.6)	1.000
	13–18 years (adolescent)	17 (77.3)	24 (77.4)	
Gender	Male	13 (59.1)	15 (48.4)	0.442
	Female	9 (40.9)	16 (51.6)	
Mother working status	Working	10 (45.5)	21 (67.7)	0.105
	Not working	12 (54.5)	10 (32.3)	
Father working status	Working	22 (100)	30 (96.8)	1.000
	Not working	0 (0)	1 (3.2)	
Parent substance use	Not using	14 (63.6)	26 (83.9)	0.013*
	Cigarette	1 (4.5)	4 (12.9)	
	Cigarettes and alcohol	7 (31.8)*	1 (3.2)	
Siblings	Yes	22 (100)	22 (71.0)	0.007*
	No	0 (0)	9 (29.0)	
Psychotropic drug use	Yes	4 (18.2)	17 (54.8)*	0.007*
	No	18 (81.8)	14 (45.2)	

Pearson Chi-square or Fisher’s exact test was used for group comparisons. *p* < 0.05 was considered statistically significant. *Indicates statistically significant subgroup differences.

The clinical characteristics of the children and adolescents with primary hypertension are summarized in Table 2. The majority of patients were receiving antihypertensive treatment, with approximately 45.5% on monotherapy and 27.3% on combination therapy. Medication adherence was assessed based on self-report obtained from the children/adolescents and their parents during face-to-face interviews. Regular medication use was reported by 54.5% of the patients. Dietary therapy had been recommended to half of the patients, among whom 36.4% demonstrated good adherence. In addition, comorbid conditions were present in 31.8% of the patients, and a positive family history was reported in 72.7% of cases.

**Table 2:** Clinical characteristics of children and adolescents with primary hypertension.

Variables	Subgroups	Primary hypertension group *n* (%)
Medication	No	6 (27.3)
	Single	10 (45.5)
	Multiple	6 (27.3)
Medication compliance	Not using	8 (36.4)
	Regularly	12 (54.5)
	Irregularly	2 (9.1)
Diet therapy	Yes	11 (50.0)
	No	11 (50.0)
Diet compliance	No treatment	11 (50.0)
	Adherent	8 (36.4)
	Poor dietary adherence	1 (4.5)
	Treatment recommended but non-adherent	2 (9.1)
Hospitalization	Yes	15 (68.2)
	No	7 (31.8)
Comorbidity	Yes	7 (31.8)
	No	15 (68.2)
Family history of hypertension	Yes	16 (72.7)
	No	6 (27.3)

Pearson or Fisher’s exact Chi-square tests were used for categorical variables.

### 3.2 Evaluation of scale scores and associated factors

When the PedsQL scores of the patient and control groups were compared (Table 3), both the parent and child Physical Health Total Scores were found to be significantly lower in the hypertension group (*p* = 0.00002 and *p* = 0.0006, respectively). Additionally, the Parent PedsQL Total Score showed a significant decrease of 6.5 points (*p* = 0.04751). Although no statistically significant differences were observed in social functioning, school functioning, or emotional functioning, all subscale scores tended to be lower in the patient group compared with the control group.

**Table 3:** Comparison of PedsQL scores between the primary hypertension and healthy control groups.

PedsQL scores (parent and child)	Primary hypertension group (*n* = 22)		Healthy control group (*n* = 31)		*p*-value
	Mean ± SD	Median (IQR)	Mean ± SD	Median (IQR)	
Parent physical health total score	78.4 ± 19.0	82.8 (30)	95.9 ± 7.4	100 (6)	0.00002*
Parent emotional functioning score	76.4 ± 17.3	77.5 (31)	78.7 ± 21.3	80 (40)	0.444
Parent social functioning score	89.3 ± 20.7	100 (14)	94.0 ± 11.7	100 (5)	0.673
Parent school functioning score	84.1 ± 17.0	85 (26)	85.8 ± 16.9	90 (25)	0.548
Parental psychosocial health total score	83.3 ± 12.9	85 (13)	86.3 ± 14.7	90 (22)	0.192
Parent PedsQL total score	82.1 ± 13.6	83 (13)	88.6 ± 11.6	91 (16)	0.04751*
Child physical health total score	80.4 ± 21.0	86 (27)	95.6 ± 8.2	100 (6)	0.0006*
Child emotional functioning score	76.1 ± 23.3	80 (28)	79.5 ± 24.2	95 (40)	0.400
Child social functioning score	82.3 ± 28.7	100 (40)	89.7 ± 13.7	100 (20)	0.780
Child school functioning score	82.7 ± 20.6	85 (26)	82.9 ± 17.7	90 (35)	0.861
Child psychosocial health total score	80.4 ± 20.4	85 (28)	84.0 ± 15.9	88 (27)	0.531
Child PedsQL total score	80.4 ± 18.1	83 (28)	86.9 ± 13.2	90 (23)	0.152

Mann–Whitney U test; *p* < 0.05 statistically significant. *Indicates statistically significant difference. PedsQL, Pediatric Quality of Life Inventory; IQR, interquartile range; SD, standard deviation.

As shown in Table 4, no significant differences were identified between the 8–12 and 13–18 age groups within the patient cohort across any PedsQL subscale (*p* > 0.05). In the control group, adolescents demonstrated lower emotional functioning scores compared with younger children, with borderline significance (*p* = 0.059). Overall, the patient group consistently exhibited lower scores across all subdomains compared with the control group; however, age category did not appear to influence these differences.

**Table 4:** Comparison of PedsQL scores between children and adolescents in the primary hypertension and healthy control groups.

Scale scores	Primary hypertension group (*n* = 22)			Healthy control group (*n* = 31)		
	Child (*n* = 5) Median (IQR)	Adolescent (*n* = 17) Median (IQR)	*p*-value	Child (*n* = 7) Median (IQR)	Adolescent (*n* = 24) Median (IQR)	*p*-value
Parent physical health total score	68.8 (27)	57.5 (28)	0.208	100 (9)	100 (3)	0.740
Parent emotional functioning score	70 (25)	80 (38)	0.503	90 (30)	80 (44)	0.373
Parent social functioning score	100 (43)	100 (15)	0.602	100 (0)	100 (9)	0.377
Parent school functionality score	85 (35)	85 (28)	0.750	90 (25)	92.5 (24)	0.591
Parental psychosocial health total score	85 (31)	85 (15)	0.783	88.3 (15)	90 (24)	0.792
Parent PedsQL total score	80.9 (30)	83.1 (15)	0.410	91.3 (14)	91.3 (18)	0.703
Child physical health total score	78.1 (27)	87.5 (31)	0.179	100 (6)	100 (10)	0.649
Child emotional functioning score	90 (33)	80.0 (28)	0.843	100 (5)	82.5 (50)	0.059
Child social functioning score	100 (23)	100 (43)	0.660	100 (10)	100 (25)	0.379
Child school functionality score	90 (18)	85 (30)	0.778	100 (40)	87.5 (34)	0.500
Child psychosocial health total score	80 (18)	90 (32)	0.582	95 (13)	84.2 (32)	0.138
Child PedsQL total score	80.3 (19)	83 (32)	0.969	94.7 (10)	87.4 (25)	0.138

Mann–Whitney U test; *p* < 0.05 statistically significant. PedsQL, Pediatric quality of life inventory.

**Figure 1:**
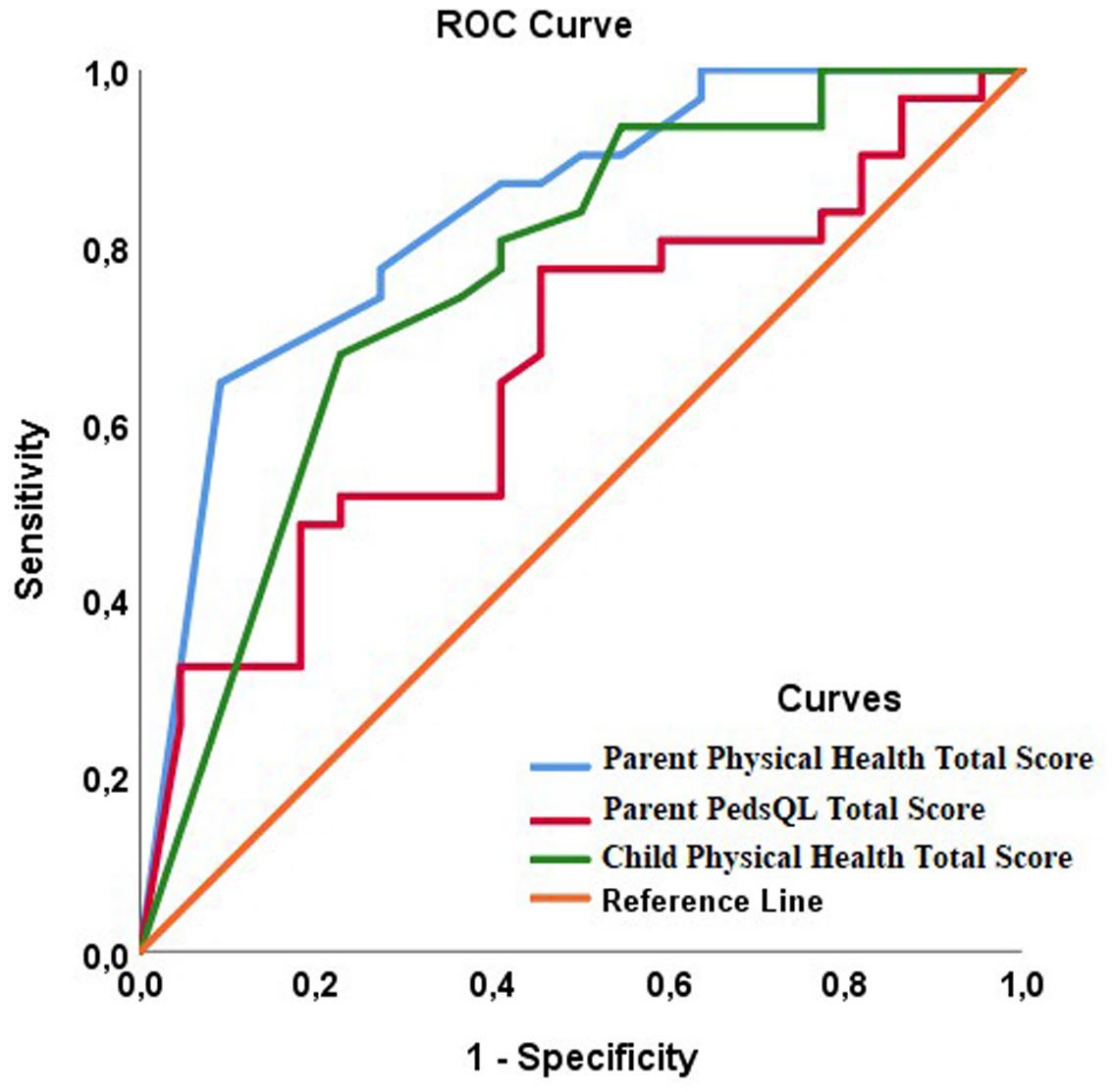
Receiver operating characteristic (ROC) curves of parameters identified as significant predictors for distinguishing patients from controls.

ROC analysis was performed exploratorily to evaluate the discriminative ability of selected PedsQL scores between the patient and control groups. According to the analysis, a Parent Physical Health Total Score below 89.1, a Parent Total PedsQL Score below 85, and a Child Physical Health Total Score below 95.3 were identified as values associated with the primary hypertension group. In addition, parent-reported physical health and total PedsQL scores demonstrated moderate discriminative ability in distinguishing children with primary hypertension from healthy controls The ROC curves are presented in Figure 1.

In the patient group, PedsQL scores from both the parent and child sections were compared according to treatment type; however, no statistically significant differences were identified between treatment categories.

When medication and diet non-adherence were examined, statistically significant differences were observed only in the diet non-adherent group (i.e., those for whom dietary treatment had been recommended but who did not comply). In this subgroup, significant reductions were found in the parent-reported Physical Health Total Score (*p* = 0.029), Social Functioning Score (*p* = 0.016), Psychosocial Health Total Score (*p* = 0.047), and overall PedsQL Total Score (*p* = 0.030). Notably, differences in PedsQL scores were observed in both the child self-report and parent proxy-report sections. When PedsQL scores were compared across BMI categories (underweight = 1, normal weight = 6, overweight = 15 patients), no statistically significant differences were observed in either the parent or child sections (*p* > 0.05).

## 4 Discussion

This study used patient- and parent-reported questionnaires to evaluate quality of life in children and adolescents diagnosed with primary hypertension. Our findings demonstrated that both parent-reported and child-reported total physical health scores were significantly lower in the hypertension group compared with the control group (*p* = 0.00002 and *p* = 0.0006, respectively). In addition, a significant 6.5-point decrease was observed in the parent-reported total PedsQL score (*p* = 0.04751).

Hypertension, one of the leading causes of end-stage renal disease in adults, is increasingly recognized as a significant health problem in childhood as well. Our patient group appeared to show demographic characteristics generally consistent with those reported in the broader pediatric primary hypertension population, particularly regarding the predominance of adolescents among affected individuals. In our study, only one patient was younger than 10 years, which supports the literature indicating that primary hypertension typically occurs after the age of ten, whereas secondary hypertension is more common in younger children (Rosner et al., 2013).

Although the prevalence of pediatric hypertension varies across geographic regions, the patient profile in our study is similar to data reported from Türkiye. For example, Mazicioglu et al. reported systolic, diastolic, and combined hypertension prevalences of 2.4, 2.5, and 1.6%, respectively, among children aged 6–18 years (Mazicioglu et al., 2010). Similarly, in a large study of 11,551 high school students aged 15–18 years, Dinç et al. found a hypertension prevalence of 3.5% and a prehypertension prevalence of 14% (Dinç et al., 2009). These findings highlight the considerable burden of elevated blood pressure among school-aged children and adolescents and underscore the clinical importance of evaluating health-related quality of life (HRQoL) in this population.

Studies focusing on quality of life in childhood are relatively limited. Nevertheless, assessing quality of life is becoming increasingly important for determining appropriate treatment strategies and managing the therapeutic process effectively. Previous research has demonstrated that chronic diseases negatively impact quality of life in children (Schwimmer et al., 2003). Regular medical follow-up, chronic medication use, dietary and lifestyle modifications, concerns regarding long-term health consequences, and disease-related psychosocial stress may contribute to impaired physical and psychosocial well-being in these patients. In addition, physical symptoms such as headache, fatigue, and reduced exercise tolerance may further negatively influence daily functioning and perceived quality of life. In a large-scale study including 139 hypertensive children aged 5–18 years, both child self-reports and parent proxy-reports demonstrated significantly lower overall HRQoL scores in the hypertensive group compared with controls, with the most pronounced differences observed in the physical and emotional functioning subscales (Petek et al., 2018). A similar pattern was observed in our study, in which the reduction in physical health scores emerged as the most prominent finding. Although statistical significance was not reached in the social and school functioning subscales, a consistent trend unfavorable to the patient group was evident across all domains.

When evaluated according to age groups, no statistically significant differences in PedsQL scores were found between the 8–12 and 13–18 year age groups in our study. This finding partially contrasts with the results reported by Petek et al., who demonstrated significantly lower social functioning scores in hypertensive children compared with hypertensive adolescents (Petek et al., 2018). This discrepancy may be attributable to methodological differences, including sample size and characteristics of the clinical population.

On the other hand, the literature also contains conflicting findings. A population-based study conducted in Germany paradoxically reported that elevated blood pressure was associated with lower psychological distress and better quality of life (Berendes et al., 2013). Adolescents with high blood pressure demonstrated higher scores in family life, self-esteem, and physical well-being, while parent reports indicated fewer emotional and behavioral problems (Berendes et al., 2013). The most likely explanation for this finding is that, due to the epidemiological and community-based design of the study, the majority of participants were probably unaware of their hypertensive status. Indeed, awareness of a chronic disease diagnosis itself is known to negatively affect quality of life independently, as physician-diagnosed hypertension may lead to anxiety, perceived restriction, and feelings of stigmatization. In contrast, our study included children with diagnosed primary hypertension who were under active clinical follow-up, and these patients demonstrated significantly lower physical health scores compared with controls. This finding likely reflects the clinical burden of chronic disease, antihypertensive medication use, and mandatory lifestyle modifications in diagnosed and treated children.

In the study conducted by McNiece et al., the rates of hypertension and prehypertension were reported to be over 30% in obese adolescent boys and between 23 and 30% in obese adolescent girl (McNiece et al., 2007). In our study, one of the patients in the patient group was underweight, six were within the normal body mass index range, and fifteen were overweight. It was found that 15 out of 22 patients (68.1%) were overweight, supporting the relationship between hypertension and obesity. However, when the effect of obesity on quality of life was examined, no statistically significant difference was found. This result is thought to be due to the small sample size.

Pediatric hypertension is not limited to long-term renal consequences; its most important effects are early cardiovascular damage and an increased risk of cardiovascular events in adulthood. Elevated blood pressure during childhood has been associated with left ventricular hypertrophy (LVH), increased carotid intima-media thickness (cIMT), arterial stiffness, and a higher risk of coronary artery disease later in life (Chung et al., 2023; Khoury and Urbina, 2018; Jacobs et al., 2022). Hypertension can lead to ocular effects such as hypertensive retinopathy, choroidopathy, and optic neuropathy. In a retrospective evaluation conducted in England, hypertensive retinopathy was reported in 28% of patients with stage 2 hypertension (Williams et al., 2013).

Our study has several limitations. The small sample size in our study limited the statistical power of the analyses. This was particularly evident in subgroup analyses, such as those involving body mass index (BMI) groups and treatment groups, where reaching statistically significant results was challenging. The single-center and cross-sectional design of the study are also important limitations. Conducting the study at a single center restricts the generalizability of the findings, while the cross-sectional design prevents causal inferences regarding the impact of hypertension on quality of life. Additionally, the majority of participants (77.4%) were adolescents aged 13–18 years, leading to an uneven distribution across age groups and limiting the comparability of outcomes by age. Furthermore, the treatment groups were not categorized based on age, which hindered the evaluation of treatment-related differences independent of age effects.

The use of a healthy control group rather than a chronic disease comparison group may have limited the ability to distinguish the specific impact of hypertension from the general burden associated with chronic disease management. Another limitation of this study is that standardized psychiatric assessments for depression or depressive symptoms were not performed in either group. Therefore, the potential confounding effects of underlying psychological conditions on health-related quality of life outcomes could not be fully controlled.

Further large-scale, multicenter studies are needed to validate these findings and to better clarify the relationship between primary hypertension and health-related quality of life in children and adolescents.

In conclusion, this study demonstrated that children and adolescents diagnosed with primary hypertension experience a significant reduction in health-related quality of life compared with their healthy peers. These findings highlight the need to integrate routine quality-of-life assessments into the clinical management of pediatric hypertension; identifying the affected domains will allow clinicians to adopt a holistic approach to treatment planning that addresses not only blood pressure control but also the psychosocial support needs of patients. Moreover, the data generated in this study lay the groundwork for future testable hypotheses—such as the mediating effects of hypertension awareness, comorbidities, target organ damage, and treatment adherence on quality of life—thereby providing an important foundation for more in-depth research in this field.

## Data Availability

The original contributions presented in the study are included in the article/supplementary material, further inquiries can be directed to the corresponding author.
